# Comparison of Swiss LithoClast Trilogy™ and Pneumatic Swiss LithoClast™ in Mini-Percutaneous Nephrolithotomy in Terms of Stone Free Rate and Complications: A Single-Center Experience From a Stone Belt Country

**DOI:** 10.7759/cureus.59829

**Published:** 2024-05-07

**Authors:** Khalid Farooq, Najma Hameed, Rizwan Ullah, Akhter Nawaz, Ikram Akhunzada, Shad Muhammad, Wajid Ali

**Affiliations:** 1 Urology, Lady Reading Hospital, Peshawar, PAK; 2 Radiology, Northwest General Hospital, Peshawar, PAK; 3 Urology, Institute of Kidney Diseases, Peshawar, PAK; 4 Nephrology, Lady Reading Hospital, Peshawar, PAK; 5 Surgery, Hayatabad Medical Complex Peshawar, Peshawar, PAK

**Keywords:** stone-free rate, pneumatic swiss lithoclast™, swiss lithoclast trilogy™, mini-pcnl, mini-percutaneous nephrolithotomy, renal calculi

## Abstract

Background

Renal calculi therapy has advanced significantly in recent years, with mini-percutaneous nephrolithotomy (PCNL) emerging as a minimally invasive treatment modality. Mini-PCNL has been subjected to several modifications to achieve the best possible outcomes and reduce morbidity. This study aimed to compare the efficacy and safety of Swiss LithoClast Trilogy™ and pneumatic Swiss LithoClast™ in managing renal stones with mini-PCNL.

Methodology

This descriptive retrospective study was conducted at the Department of Urology, Lady Reading Hospital, from January 1, 2023, to December 31, 2023. A record of male and female patients aged more than 18 years who underwent mini-PCNL for renal stones was retrieved. The following two groups of patients were created: group A (n = 25) mini-PCNL with Swiss LithoClast Trilogy™ and group B (n = 26) mini-PCNL with pneumatic Swiss LithoClast™. The efficacy and safety profile of both groups was compared.

Results

A total of 51 patients were enrolled, with 25 in group A and 26 in group B. Groups A and B had mean ages of 45.2 and 47.5 years, respectively. Male participants outnumbered females in both groups, 72% (n = 18) in group A and 77% (n = 20) in group B. Group A had a mean stone size of 15.8 mm, and group B had a mean stone size of 16.5 mm. Stone-free rate on postoperative day one was 88% (n = 22) in group A and 84.6% (n = 22) in group B, with no statistically significant difference (p > 0.05). At the end of three months, 96% of participants in group A and 84.6% of patients in group B were found to be free of stones, and the difference between the two groups was not statistically significant (p > 0.05). Intraoperative hemorrhage occurred in 12% (n = 3) of group A and 15.4% (n = 4) of group B patients, with no significant difference (p > 0.05).

Conclusions

There were no significant differences in stone-free rates, complication rates, or intraoperative/postoperative complications between mini-PCNL with Swiss LithoClast Trilogy™ or Pneumatic Swiss LithoClast™.

## Introduction

Kidney stone disease is a significant worldwide health issue, impacting a considerable proportion of the population [1]. The treatment approaches for renal calculi have made significant advancements over the years. Minimally invasive techniques are rapidly replacing the conventional management approach. Mini-percutaneous nephrolithotomy (mini-PCNL) has emerged as a beneficial and effective therapy [2-4]. This minimally invasive procedure, distinguished by its decreased tract size and reduced morbidity, provides an efficient strategy for addressing renal stones [5,6]. Mini-PCNL has also been subjected to several modifications over the years to achieve the best possible results and limited complications.

Switzerland, famous for its advancements in medical innovation, has produced lithotripsy instruments specifically intended to improve the effectiveness of stone fragmentation during mini-PCNL procedures [7]. Among them, the Swiss LithoClast Trilogy™ and Pneumatic Swiss LithoClast have attracted attention for their potential influence on stone-free rates and procedural safety. However, discrepancies in equipment functionality may occur, primarily when used in different clinical environments, such as regions with a high incidence of kidney stones, sometimes referred to as stone belt countries [8,9].

This study primarily examined the outcomes of the stone-free rate and the occurrence of complications in a stone belt country. While the majority of the studies conducted on similar topics come from centers with better resources, none of these studies have compared the Swiss LithoClast Trilogy with Pneumatic Swiss LithoClast. This is the first of its kind study comparing the two modalities in a single study. Swiss LithoClast Trilogy is a lithotripter that utilizes controlled ultrasonic shockwaves for stone fragmentation and simultaneously sucks out the fragments through the suction channel. The Pneumatic Swiss LithoClast is a combination of pneumatic and ultrasonic lithotripter. The pneumatic part primarily fragments the stone while the ultrasonic part fragments it further and simultaneously sucks it out.

## Materials and methods

A descriptive retrospective study was conducted at the Department of Urology at Lady Reading Hospital Peshawar from January 1, 2023, to December 31, 2023. Ethical approval was obtained from Lady Reading Hospital Medical Teaching Institution Institution Review Board (IRB) (approval number: 29/LRH/MTI). Male and female patients in the age range of 18 to 70 years diagnosed with renal calculi on ultrasound or unenhanced CT of the kidneys, ureters, and bladder (KUB) who underwent mini-PCNL were enrolled. Patients with a prior history of renal surgery, pregnant females, chronic kidney disease, and severe cardiopulmonary disease were excluded. Patients with incomplete records were also excluded.

Operational definitions

Stone-Free Rate

The stone-free rate was defined by the presence of leftover fragments of stone measuring 4 mm or less confirmed on imaging (ultrasound) or CT KUB performed on the first postoperative day.

Complications

Complications were broadly classified as intraoperative and postoperative complications. Intraoperative complications included intraoperative bleeding measured by comparing the preoperative hemoglobin level with the hemoglobin level measured on the first postoperative day. A drop in hemoglobin level of 1 g/dL or more was considered significant. Postoperative complications were measured using the Clavien-Dindo classification system [10]. Patients with grade I or II Clavien-Dindo complications such as fever, emesis, and minor pain were grouped as minor complications while grade III to V complications such as renal dysfunction, multiorgan failure, or any other complications requiring intervention under local or general anesthesia were grouped as major complications.

The records of 51 patients were retrieved from the hospital’s Health Management Information System. Preoperative demographics of the participants were recorded, including age, gender, and body mass index (BMI). Stone characteristics that were recorded included the laterality of the stone, stone size, and composition. All procedures were performed under general anesthesia. After proper prone positioning, a small incision was made on the flank of the affected side. A tract was created, and a PCNL access needle was introduced with sheath sizing ≤18 Fr. The stone was accessed, and fragmentation was performed using Swiss LithoClast Trilogy or Pneumatic Swiss LithoClast. Patients were grouped as mini-PCNL with Swiss LithoClast Trilogy (group A) or mini-PCNL with Pneumatic Swiss LithoClast (group B) based on the fragmentation instrument. Stone fragmentation in group A was performed with Swiss LithoClast Trilogy which is a lithotripter that generates ultrasonic shockwaves to fragment large stones and simultaneously sucks them out. Fragmentation and suction in group B were performed using Pneumatic Swiss LithoClast which is a combination of a pneumatic and ultrasonic lithotripter, with the pneumatic part primarily fragmenting the stone while the ultrasonic part fragmenting it further and simultaneously sucking it out. Ultrasound or CT KUB findings performed on the first postoperative day to confirm stone-free status were noted. Operative time and intraoperative blood loss were determined. Blood hemoglobin was measured on the first postoperative day and compared with preoperative hemoglobin to measure intraoperative blood loss. The Clavien-Dindo classification was used to measure the secondary outcomes [10].

Data were analyzed using SPSS version 25 (IBM Corp., Armonk, NY, USA). Means ± SD or median interquartile range was computed for quantitative data after applying the Wilk normality test. Frequencies and percentages were recorded for qualitative variables. Both groups were compared for stone-free rate and complications. Categorical variables were compared using the chi-square or Fisher exact test, while continuous variables were compared using the independent-sample t-test or Mann-Whitney U test. P-values ≤0.05 were considered statistically significant.

## Results

This study included 51 patients, 25 in group A, who underwent Swiss LithoClast Trilogy, and 26 in group B, who underwent Pneumatic Swiss LithoClast. Group A had a mean age of 45.2 years, and group B had a mean age of 47.5 years. The male population exceeded the female population in both groups, comprising 72% (n = 18) and 77% (n = 20), respectively. The gender distribution exhibited no significant variation between the two groups. Group A had an average BMI of 26.4 kg/m^2^, while group B had an average BMI of 27.1 kg/m^2^. The demographic of participants was comparable, as shown in Table 1.

**Table 1:** Demographics of the study participants. P-values ≤0.05 were considered statistically significant. SD = standard deviation; BMI = body mass index

Characteristic	Group A (Swiss LithoClast Trilogy™)	Group B (Pneumatic Swiss LithoClast)	P-value
Age (years), mean ± SD	45.2 ± 8.3	47.5 ± 9.1	0.342
Gender			
Male	18 (72%)	20 (77%)	0.686
Female	7 (28%)	6 (23%)	
Stone side			
Left	13 (52%)	15 (58%)	0.682
Right	12 (48%)	11 (42%)	
BMI (kg/m²), mean ± SD	26.4 ± 3.1	27.1 ± 2.9	0.267

**Figure 1:**
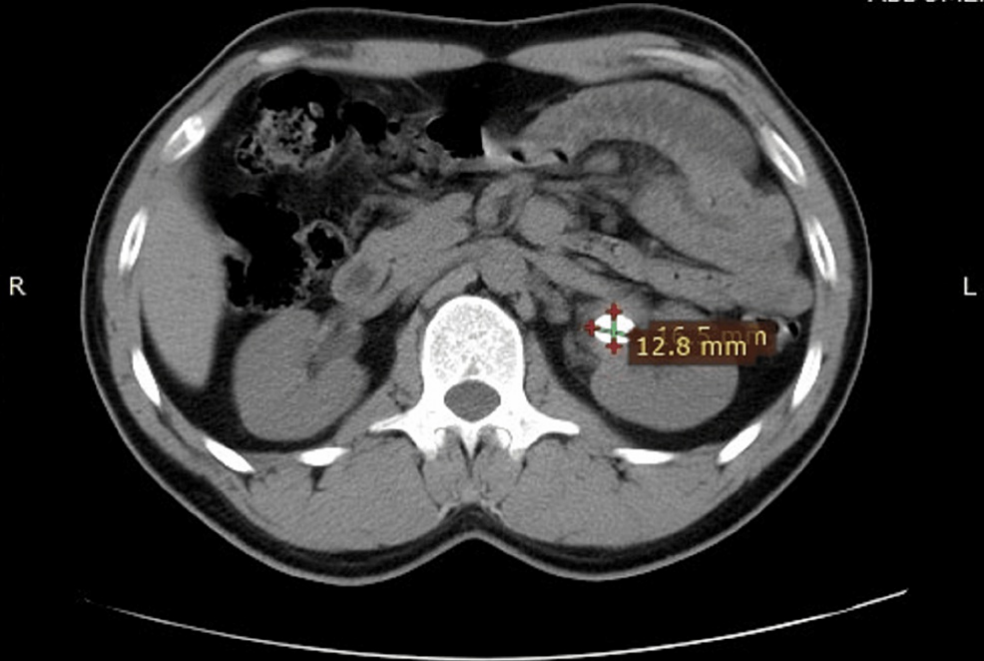
Preoperative CT KUB image showing a calculus in the left kidney (coronal view). CT KUB = computed tomography of the kidneys, ureters, and bladder

**Figure 2:**
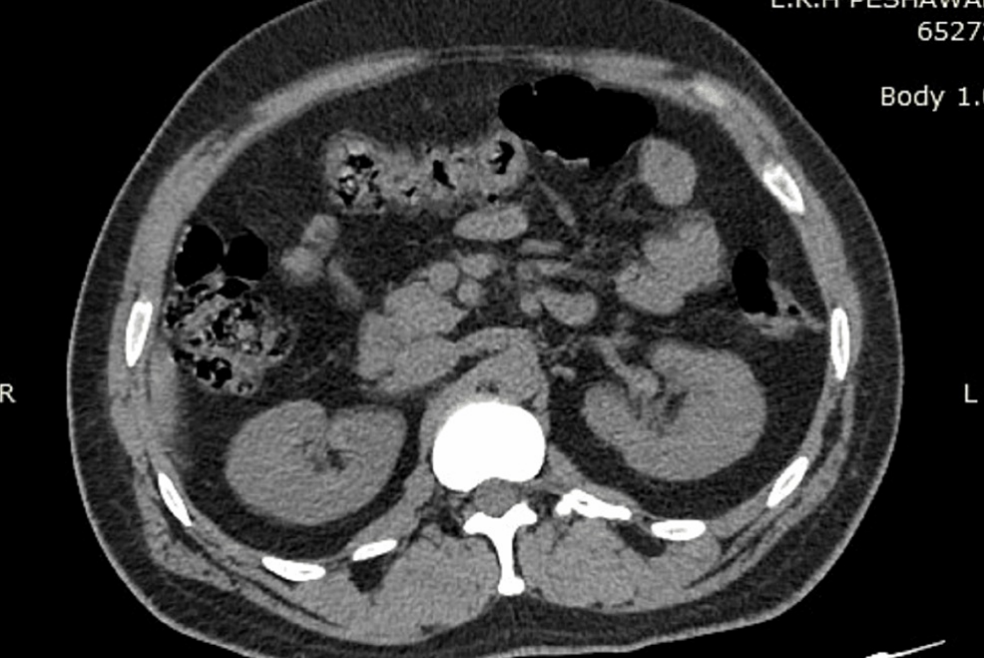
Postoperative CT KUB image (stone clearance achieved using Swiss LithoClast Trilogy). CT KUB = computed tomography of the kidneys, ureters, and bladder

**Figure 3:**
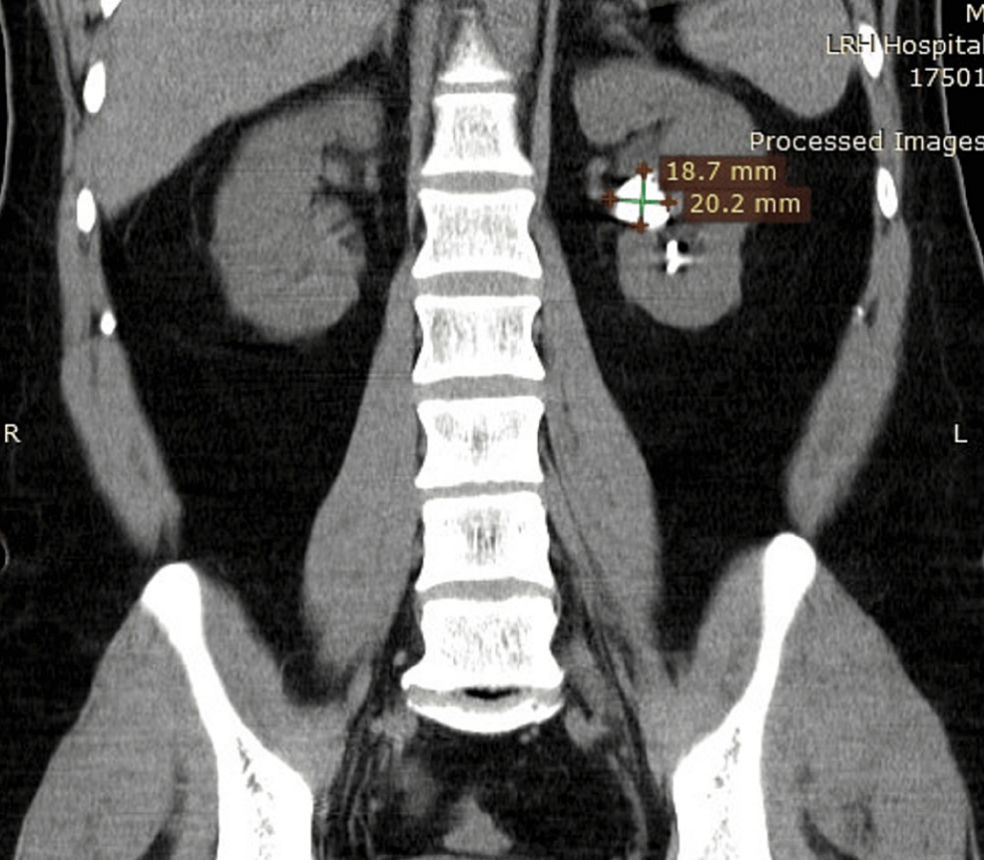
Preoperative CT KUB image showing a large left kidney stone. CT KUB = computed tomography of the kidneys, ureters, and bladder

**Figure 4:**
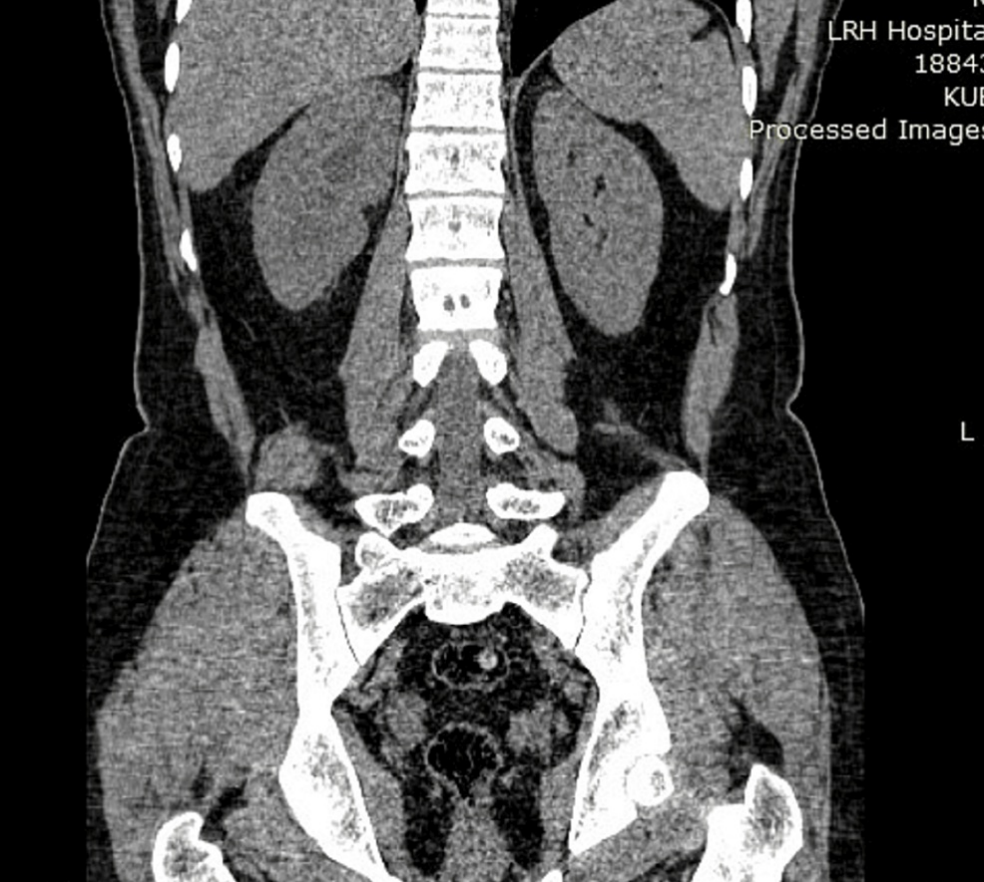
Postoperative CT KUB image (stone clearance achieved using Pneumatic Swiss LithoClast). CT KUB = computed tomography of the kidneys, ureters, and bladder

The mean stone size in groups A and B was 15.8 mm and 16.5 mm, respectively. Both categories mainly consisted of stones composed of either calcium oxalate or calcium phosphate. There was no noticeable change in the composition of the stones between the two groups. The average tract size for the mini-PCNL treatment was similar in both group A and group B, with group A having a size of 16.6 Fr and group B having a size of 16.2 Fr. Group A had an average operation time of 75.4 minutes and group B had an average procedure time of 78.2 minutes. Figure 1 and Figure 2 show the preoperative and postoperative CT KUB images, respectively, of a patient whose stone clearance was achieved using the Swiss LithoClast Trilogy. Similarly, Figure 3 and Figure 4 show the preoperative and postoperative CT KUB images, respectively, of a patient whose stone clearance was achieved using the Pneumatic Swiss LithoClast.

The characteristics of the stones of the mini-PCNL procedure were similar across the two groups, as shown in Table 2.

**Table 2:** Stone characteristics and mini-PCNL procedure details. P-values ≤0.05 were considered statistically significant. SD = standard deviation; PCNL = percutaneous nephrolithotomy

Characteristic	Group A (Swiss LithoClast Trilogy™) n = 25	Group B (Pneumatic Swiss LithoClast™) n = 26	P-value
Stone size (mm), mean ± SD	15.8 ± 3.2	16.5 ± 3.8	0.421
Stone composition			
Calcium oxalate	20 (80%)	18(69%)	0.189
Calcium phosphate	05 (20%)	08 (31%)	
Tract size (Fr), mean ± SD	16.6 ± 1.4	16.2 ± 1.8	0.563
Operation time (minutes), mean ± SD	75.4 ± 12.5	78.2 ± 14.3	0.327

The rates of complete absence of stones at day one and three months post-surgery were compared between the two groups. On the first day after surgery, 88% (n = 22) of patients in group A and 84.6% (n = 22) of patients in group B were stone-free. There was no notable difference between the two groups. At the end of three months, 96% of participants in group A and 84.6% of patients in group B were found to be stone-free. There was a slight inclination toward a greater stone-free rate in group A, but this difference did not reach statistical significance. The complication rates of both groups were also compared using the Clavien-Dindo classification. Minor complications were observed in four (16%) and five (19%) patients in groups A and B, respectively, while significant complications were observed in two (8.0%) and three (11.5%) patients in groups A and B, respectively. However, it is essential to note that this disparity did not reach statistical significance, as illustrated in Table 3.

**Table 3:** Stone-free rates at postoperative day one and three months. P-values ≤0.05 were considered statistically significant.

Stone-free status	Group A (Swiss LithoClast Trilogy™)	Group B (Pneumatic Swiss LithoClast)	P-value
Postoperative day 1, n (%)	22 (88%)	22 (84.6%)	0.725
Postoperative month 3, n (%)	24 (96%)	22 (84.6%)	0.171
Clavien-Dindo classification complication rates			
I (minor complications), n (%)	4 (16%)	5 (19%)	0.734
II (major complications), n (%)	2 (8%)	3 (11.5%)	0.621

Table 4 presents a comparison of the problems that occurred during surgery and after surgery in both groups. The incidence of intraoperative bleeding was 12% (n = 3) in group A participants and 15.4% (n = 4) in group B participants, with no statistically significant disparity between the two groups. Postoperative fever occurred in 8% (n = 2) of individuals in group A and 11.5% (n = 3) in group B, with no statistically significant difference between the two groups. Additional issues, such as urinary tract infection or injury to the ureter, were seen in 4% (n = 1) of individuals in group A and 7.7% (n = 2) of individuals in group B, as shown in Table 4.

**Table 4:** Comparison of intraoperative and postoperative complications. P-values ≤0.05 were considered statistically significant.

Complication type	Group A (Swiss LithoClast Trilogy)	Group B (Pneumatic Swiss LithoClast)	P-value
Intraoperative bleeding, n (%)	3 (12%)	4 (15.4%)	0.652
Postoperative fever, n (%)	2 (8%)	3 (11.5%)	0.731
Other complications, n (%)	1 (4%)	2 (7.7%)	0.589

## Discussion

Management of renal stones has been revolutionized from conventional open surgery to advanced minimally invasive techniques employing various tools for removing stones such as Swiss LithoClast Trilogy and Pneumatic Swiss LithoClast [11]. The performance of these techniques has been a matter of debate over the years. This is a first-of-its-kind study comparing the two concerning stone-free rates and complications. Our study results suggest no significant differences in stone-free rates, complication rates, or intraoperative and postoperative issues between the two groups using different pneumatic lithotripters for mini-PCNL. The findings are consistent with other studies investigating several lithotripters for mini-PCNL. In a study performed by Cauni et al. to assess the performance of Swiss LithoClast Trilogy, the stone-free rate was 82.1% at the end of the procedure and 89.3% one month after the procedure which is comparable to our results (88% on the first postoperative day and 96% after three months of surgery). Similarly, in our study, in the Swiss LithoClast Trilogy group, minor complications occurred in 16% of patients, and major complications occurred in 8% of patients, while in the study performed by Cauni et al., the overall complication rate was 17.9% which is also comparable to our complication rate [12]. However, our research revealed a discernible inclination toward a greater stone-free rate in group A (utilizing the Swiss LithoClast Trilogy™) three months after the surgical procedure.

In a study by Cho et al., the overall stone-free rate achieved with Pneumatic LithoClast was 85.2% and the overall complication rate was 11.4%. The success rate of their study is similar to ours with an 84.6% success rate. However, the complication rate is much lower than in our study. The lower rate of complications may be attributed to the shorter follow-up period (four weeks) in their study compared to the prolonged follow-up (12 weeks) in our study [13]. In another study by Haghighi et al. comparing ultrathin PCNL with standard PCNL utilizing Pneumatic LithoClast for stone removal, the success rate varied from 93.58% to 94.6% with complications rate ranging between 5.7% and 11.4%. Apart from using different caliber endoscopes which may have led to a higher success rate, the mean stone size in their study was lower than our study. The smaller caliber stones could have led to a lower complication rate compared to our patients [14].

Limitations

A single-center design, small sample size, and retrospective study design are some of the major limitations of this study. All these factors limit the generalizability of the study. However, our study provides a platform for prospective studies on this important topic.

## Conclusions

The study found no significant difference in stone-free rates, complication rates, or intraoperative/postoperative problems between mini-PCNL with Swiss Lithoclast Trilogy or Pneumatic Swiss Lithoclast for mini-PCNL. At postoperative day one, the stone-free rates were comparable between the two groups (group A: 88%, mini-PCNL with Swiss LithoClast Trilogy; group B: 84.6%, mini-PCNL with Pneumatic Swiss Lithoclast). Similarly, stone-free rates showed no significant difference between the two groups three months after surgery. However, stone-free rates were numerically better with Swiss LithoClast Trilogy than Pneumatic Swiss LithoClast at postoperative day one and three months. The better performance of the Swiss LithoClast Trilogy could be attributed to its better design. Large multicenter prospective studies are needed to provide more reliable results.
